# Advanced Image Analysis Methods for Automated Segmentation of Subnuclear Chromatin Domains

**DOI:** 10.3390/epigenomes6040034

**Published:** 2022-10-05

**Authors:** Philippe Johann to Berens, Geoffrey Schivre, Marius Theune, Jackson Peter, Salimata Ousmane Sall, Jérôme Mutterer, Fredy Barneche, Clara Bourbousse, Jean Molinier

**Affiliations:** 1 Institut de Biologie Moléculaire des Plantes du CNRS, 67000 Strasbourg, France; 2 Institut de Biologie de l’Ecole Normale Supérieure (IBENS), Ecole Normale Supérieure, Centre National de la Recherche Scientifique, Inserm, Université PSL, 75230 Paris, France; 3 Université Paris-Saclay, 91190 Orsay, France; 4 FB 10 / Molekulare Pflanzenphysiologie, Bioenergetik in Photoautotrophen, Universität Kassel, 34127 Kassel, Germany

**Keywords:** nucleus, chromocenters, microscopy, automated segmentation, deep-learning

## Abstract

The combination of ever-increasing microscopy resolution with cytogenetical tools allows for detailed analyses of nuclear functional partitioning. However, the need for reliable qualitative and quantitative methodologies to detect and interpret chromatin sub-nuclear organization dynamics is crucial to decipher the underlying molecular processes. Having access to properly automated tools for accurate and fast recognition of complex nuclear structures remains an important issue. Cognitive biases associated with human-based curation or decisions for object segmentation tend to introduce variability and noise into image analysis. Here, we report the development of two complementary segmentation methods, one semi-automated (*iCRAQ*) and one based on deep learning (*Nucl.Eye.D*), and their evaluation using a collection of *A. thaliana* nuclei with contrasted or poorly defined chromatin compartmentalization. Both methods allow for fast, robust and sensitive detection as well as for quantification of subtle nucleus features. Based on these developments, we highlight advantages of semi-automated and deep learning-based analyses applied to plant cytogenetics.

## 1. Introduction

In the last decade, visualization of cellular structures has benefited major technical advances in cytochemistry and microscopy, allowing for 2D and 3D analyses at an unprecedented resolution of cellular and subcellular structures, such as organelles [1,2], cytoskeleton [3], extra cellular vesicles [4], stress granules [5] and chromatin subnuclear organization [6,7,8,9,10]. Increasing interest in chromatin-based regulation of DNA-related processes, such as transcription, replication and repair, has led to the development of a large repertoire of tools enabling qualitative and quantitative image analyses of nuclear organization. Cytogenetics studies notably allow for determining how chromosomes are structured in the cell nucleus. For example, the distribution of large chromatin domains and their possible aggregation as conspicuous structures called chromocenters, visible in species such as *Arabidopsis thaliana* [11] and *Mus musculus* [12], can be revealed by 4′,6-Diamidino-2-phenylindol (DAPI). Improvements in cytogenetic techniques and microscopic image acquisition generate large high-quality image sets that require automation or semi-automation for reliable and accurate interpretation. Furthermore, open-source software, web-assisted applications and plugins are increasingly developed and improved to assist or automatize the detection of nuclear substructures through intensity thresholding, edge detection and mathematical image transformation [13,14,15,16], including several automated tools developed for plant chromatin architecture (NucleusJ [6], NucleusJ2.0 [17], and NodeJ [18]. However, segmentation of high complexity structures exhibiting irregular shapes or intensities still remain challenging for samples from peculiar tissues. In addition, manual segmentation can be seen as the golden standard, but any decisions made by the user can be associated with cognitive biases and carry the risk of distorting the conclusion of an otherwise well-designed experiment. Accuracy of user performance likely differs from one laboratory or experimentalist to another, especially when analyzing large datasets. Additionally, cognition of each user may fluctuate depending on instrumentation, physical and cognitive biases [19]. Consequently, reducing the need for human decision at each step of the image analysis process is a critical challenge for experimental reproducibility and accuracy [20].

Recently, deep learning (DL) tools have opened new perspectives for image analysis. DL is a category of machine learning, using training datasets (i.e., images) that will feed an artificial neural network, allowing for a task of interest to be automatedly performed. DL techniques are typically based on manually segmented datasets (training and validation datasets) that are also prone to follow human biases. DL is useful to reduce inter-operator variability and to automate the resolution of certain imaging problems.

When applied to image analysis, DL can outperform classical methods in image classification [21] and denoising [22] and complex segmentation [23].

Unfortunately, despite their added value, DL-based image analysis approaches remain hardly accessible to many potential users lacking programming expertise. Only recently, the development of user-friendly open-source tools has democratized the use of DL and was recognized as an essential objective by the scientific community [24,25,26]. 

In plant biology, increasing efforts are devoted to develop DL-based tools for species identification [21], phenotypic analysis of aerial parts [27], roots [28], cells [29] and analysis of organellar morphology [30]. As the field of plant epigenetics and 3D genomics develops, interest in automated detection of nuclei and of prominent subnuclear structures such as chromocenters in microscopy images is rapidly growing [7,31]. In interphase nuclei of most Arabidopsis organs, chromocenters are formed by the coalescence between centromeres and other “heterochromatic” repeat-rich chromosomal domains such as transposable elements (TEs) and pericentromeric and sub-telomeric nucleolar organizing regions (NORs) into highly condensed 8-to-10 conspicuous foci [10,32]. Organization and morphologies of nuclei and chromocenters undergo major changes during several biological transitions such as cell differentiation [33], cell division [34], developmental switches [35,36] and upon exposure to environmental stresses [37,38] with potentially major consequences on nucleus activities. Automated characterization of nucleus organization also constitutes a relevant and timely asset to enable short to large-scale nuclear phenotype screening of mutations and conditions affecting developmental and environmental cellular responses. 

Here, we present two complementary user-friendly tools to segment *A. thaliana* nuclei and chromocenters acquired using confocal imaging. First, we developed a semi-automated ImageJ macro [39] that we called Interactive Chromocenter Recognition and Quantification (*iCRAQ*; [40]) for the purpose of facilitating nucleus and chromocenter segmentation that reduces inter-user variability while enabling visual validation of each segmented object by the user. *iCRAQ* relies on simple heuristics to guide the user during nucleus and chromocenter segmentation and accepts user input for manual curation of image segmentation. These validation and curation steps are particularly necessary when contaminants are present (debris, vessels or plastids), which can otherwise lead to misannotation as nuclei by automated tools. Second, we present *Nucl.Eye.D* [41], a DL-empowered automated nucleus and chromocenter segmentation tool. *Nucl.Eye.D* has been written by biologists for biologists and is conceptualized in a way that it can be used and adapted with low programming knowledge. Combining these two tools address application problems in DL-based modeling implementation such as the production of training repertoires of annotated images (obtained here using *iCRAQ*) and the capacity to accurately analyze highly variable image objects.

We first compared inter-user variability between manual or *iCRAQ* semi-automated segmentation methods. We then tested the spectrum of nuclear phenotypes that can reliably be analyzed with both tools, using images of Arabidopsis cotyledon nuclei in conditions and genotypes that trigger massive variations of nucleus size and/or chromocenter formation such as dark-grown seedlings [35] and *decreased DNA methylation 1* (*ddm1*) mutant plants [42]. Both sample types are characterized by extensive heterochromatin relaxation of heterochromatin, which is then scattered in poorly defined foci often hardly amenable to automated segmentation [35,43]. The image set of DAPI-stained nuclei developed for this study therefore brings the advantage of containing a well-described duality of nuclear phenotypes that allows for testing both the sensitivity and accuracy of segmentation methods. Taken together, we documented the issues associated with human decision making and developed two segmentation tools, *iCRAQ* and the DL-based tool *Nucl.Eye.D*, which are also readily usable through Google Colab environment [41].

## 2. Results and Discussion

### 2.1. iCRAQ: A Plug-In Assisted Tool for Segmentation of Nucleus and Chromocenters

**Figure 1:**
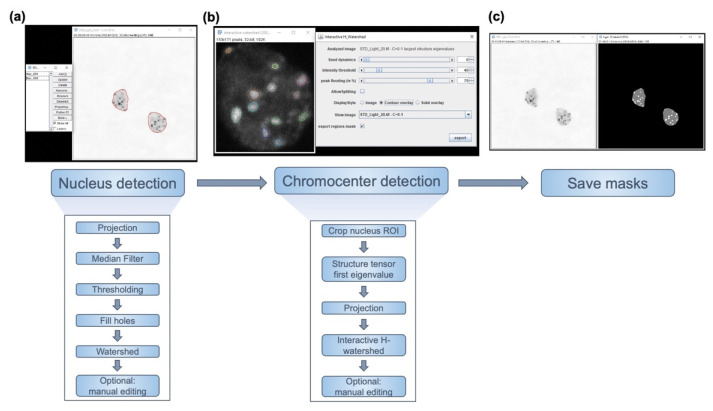
*iCRAQ* workflow. (**a**) *iCRAQ* first step is to detect nuclei in a 2D projection of z-stack images based on an intensity thresholding (resolving touching nuclei through a watershed step). Two examples of segmented nuclei are shown as regions of interest (ROIs) outlined in red. (**b**) A region surrounding each nucleus is defined and cropped from the original stack. For each single-nucleus stack, the first eigenvalue of the structure tensor is calculated at each pixel in the image stack and projected in z. This projected image is segmented interactively via the H-watershed plugin. (**c**) The final result is an image mask with three levels of gray: black for the image background, gray for the nuclear interior outside of chromocenters, and white for chromocenters. See Materials and Methods for details.

In order to minimize inter-user variability and human decision making in the process of nucleus and chromocenter segmentation, we developed an ImageJ macro: *iCRAQ* [40]. *iCRAQ* provides semi-automatic segmentation assistance, detecting nuclei and chromocenters from z-stack images acquired by confocal microscope (see materials and methods for details). Depending on the image quality, nucleus segmentation is either performed automatically using a minimum cross entropy thresholding method [44], by manual thresholding, or by drawing the nucleus outline with the ImageJ freehand selection tool. Chromocenter segmentation is performed via the H-watershed ImageJ plugin with manual intervention to attain the optimal segmentation (Figure 1; [40]). For both nuclei and chromocenters, wrongly detected objects can be individually removed or manually added with the freehand selection tool.

To test *iCRAQ* performance, three users proficient in image analysis of Arabidopsis nuclei independently segmentated nuclei and chromocenters in a contrasted set of more than 50 cotyledon nuclei from dark- and light-grown seedlings (hereafter referred to as the Dark/Light set) [35]. For comparison, manual segmentation of the Dark/Light set using ImageJ [39] was also independently performed by the three users. Both methods produce binary masks of nuclei and chromocenters that are either used either directly for inter-user comparisons or to quantify several nuclear features including the number of visible chromocenters (CC) per nucleus, the nuclear area, the relative CC area (area of each CC per nucleus), the heterochromatin fraction (HF, i.e., sum of all chromocenters’ area relative to the whole nucleus area), the relative heterochromatin intensity (RHI, i.e., chromocenter-to-nucleus mean intensity ratio) and the relative heterochromatin fraction (RHF, i.e., the proportion of stained DNA present in chromocenters; see Materials and Methods for more detail).

**Figure 2:**
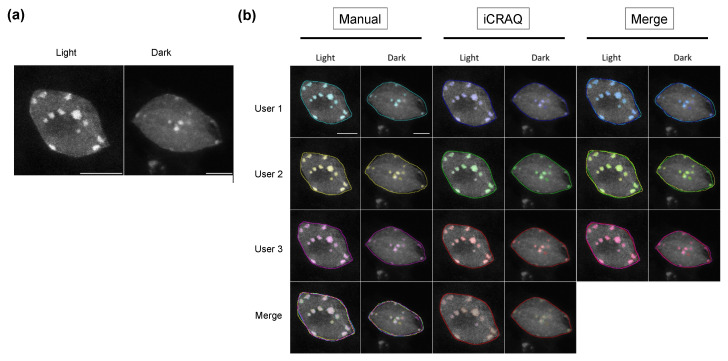
Segmentation of nuclei and chromocenters by three different users. (**a**) Representative DAPI stained nuclei from the Light/Dark set. (**b**) ROIs resulting from manual or *iCRAQ* segmentation methods. Merged images show the overlap between segmentation masks, comparing differences between users and methods. Scale bar = 5 μm.

**Figure 3:**
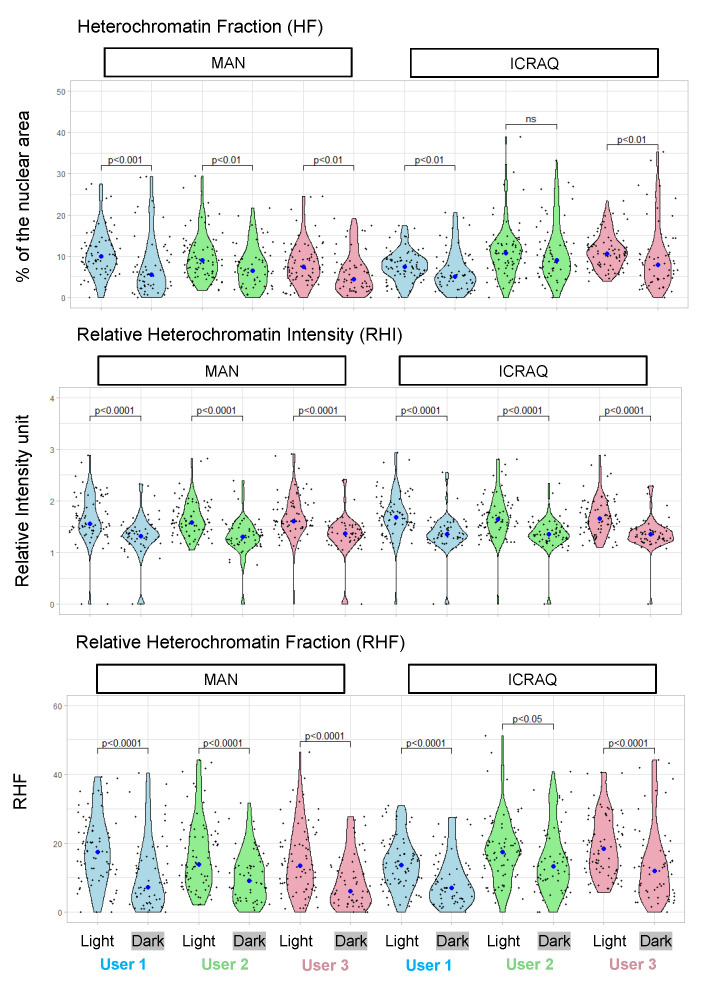
Comparison of heterochromatin parameters quantified by different users and segmentation methods. Violin plots illustrating the distribution of heterochromatin fraction (HF), relative heterochromatin intensity (RHI) and relative heterochromatin fraction (RHF) in nuclei from the Light/Dark set. Each dot represents the measure for one nucleus. The big dot shows the median value. Displayed *p* values were obtained by the Mann–Whitney–Wilcoxon test (n > 50 per condition).

As shown with representative nuclei from the Dark/Light set (Figure 2A), inter-user differences in nucleus and chromocenter manual segmentation can be observed when performing manual segmentation. Whereas segmentations only slightly differ at the edge regions of chromocenters of the light condition wherein these sub-nuclear domains form conspicuous foci, users do not always agree on chromocenter segmentation for the dark condition characterized by more complex heterochromatic structures and less contrasted patterns (Figure 2B). However, trends obtained by all three users were in agreement with previous studies reporting a significantly lower HF, RHI and RHF in dark than in light conditions (Figure 3) [35]. Noteworthy, depending on the user, the mean RHF for Light and Dark nuclei ranges between 15–18% and 8–11%, respectively. With regard to nucleus area and relative CC area, both features display variable changes under light and dark conditions depending on the user (Figure S1). This sheds light on inter-user variability being a significant issue potentially leading to inappropriate conclusions. In addition, for all users, RHF also differed between manual and *iCRAQ* segmentation (Figure 3). In addition, these comparative analyses put emphasis on the fact that measures of heterochromatin organization should always be expressed as relative to an internal control (i.e., wild-type nuclei originating from control growth condition) as absolute values for the different parameters vary between users while the trends are always conserved (Figure 3).

**Figure 4:**
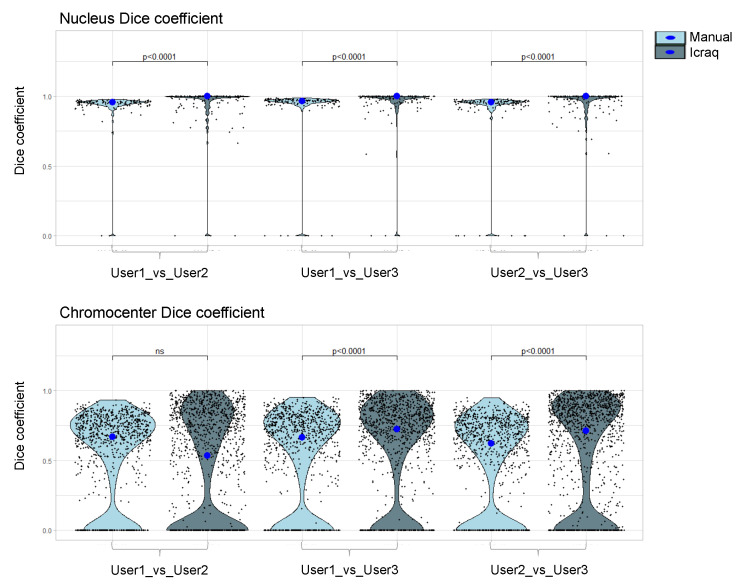
Dice coefficient between segmentation masks from three different users using manual or *iCRAQ* segmentation. All objects from segmentation masks are compared between users for both segmentation methods. A Dice coefficient of 1 signifies that the object was identically segmented by User_A and User_B. Statistical comparison was performed in between the segmentation methods according to the Mann–Whitney–Wilcoxon test. Each dot represents the measure for one object. The big dot shows the median value. n = 50 for nuclei and n > 400 for chromocenters.

In order to test and compare the *iCRAQ* segmentation tool for inter-user variability, we calculated the Dice coefficient to measure similarities between binary masks obtained using manual and *iCRAQ* segmentation by pairs of users (Figure S2). For each pairwise comparison, the nucleus Dice coefficient significantly increased with *iCRAQ* compared to manual segmentation (Figure 4). In parallel, Dice coefficient for chromocenter segmentation showed an increased inter-user variability as compared to nucleus segmentation, also improved by *iCRAQ* for two of the three users (Figure 4). These observations highlight that cognitive biases [21] occurring during manual segmentation induce high variability, while assistance, using *iCRAQ,* can improve the reproducibility of object recognition. Taken together, these results show that *iCRAQ* tends to reduce the inter-user variability, a benefit that remains dependent on each user tendency for manual readjustment of the segmentation. Hence, while *iCRAQ* necessitates significant manual intervention to define H-watershed thresholds and include or remove individual objects, it provides a robust and accurate semi-automated tool for nucleus and chromocenter quantification enabling both individual analyses and the production of training datasets.

Reproducibility also suffers from the objectiveness of the discriminative features of the object of interest. Accordingly, while all users agreed on the definition of a chromocenter as distinct bright foci inside the nucleus (Figure 2A), they may differ on their definition of “distinct” and “bright”, thus leading to discordant segmentation. Furthermore, *iCRAQ*-assisted segmentation guides segmentation toward objects that meet criteria measurable by the software but which only approximate the objects’ distinctive features visually recognized by the users.

### 2.2. Nucl.Eye.D: A Fully Automated Deep Learning Pipeline for Segmentation of Nucleus and Subnuclear Structures

**Figure 5:**
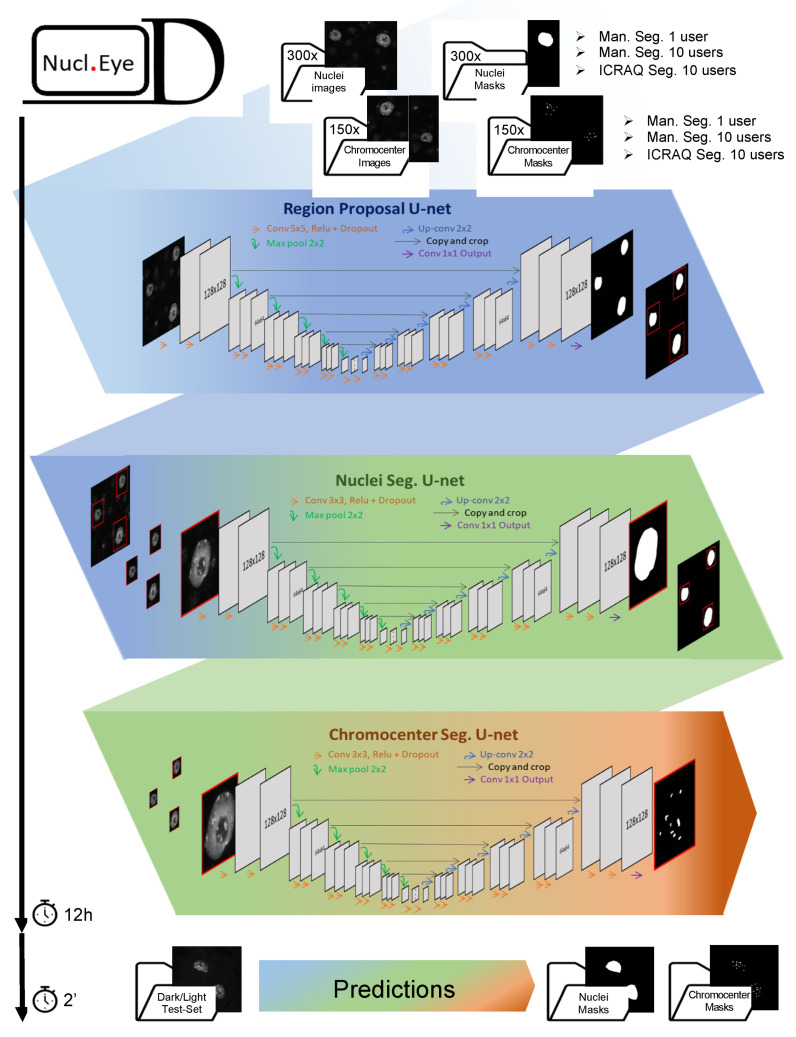
The *Nucl.Eye.D* pipeline. *Nucl.Eye.D* consists of three successive neuronal U-networks. In a first step, the nucleus training set is fed into a *Region Proposal U-Net Model* that aims at making a raw trim window of regions in the image containing a single nucleus. In a second step, the predicted bounding boxes are used to produce small image fragments, which will in turn feed the *Nucleus Segmentation U-Net model*. This second model precisely predicts the boundaries of each nucleus previously identified by the *Region Proposal U-Net Model*. Finally, nuclei from the chromocenter training set are successively segmented using the *Region Proposal U-Net Model* and *Nucleus Segmentation U-Net model*, providing small image fragments that will be used to train the *Chromocenter Segmentation U-Net model*. To prevent model overfitting, which can be a consequence of small training datasets, the pipeline includes optional data-augmentation steps. The training period takes around 12 h for detection of both nuclei and chromocenters using a training set composed of 300 and 150 images, respectively. Prediction takes about 2 s per Dark/Light test-set image.

To enable fast and high-throughput image analysis and to overcome low segmentation reproducibility due to intra- and inter-user variability, we set up a fully automated DL-based tool for nucleus and chromocenter segmentation: *Nucl.Eye.D* ([41]; Figure 5). Importantly, this tool was developed to reproduce realistic average lab conditions, such as the availability of limited training image datasets displaying inter-user diversity in sample preparation and image acquisition. The script includes all necessary codes and explanations for any user with basic programming skills to easily train his/her own model with his/her own images in case the provided pre-trained model is not fitted for the intended use [41]. In this example, training was performed using two sets of 300 and 150 images (with an average of 5 nuclei/image) for nucleus and chromocenter segmentation, respectively. Considering that segmentation by a DL-based tool can only be efficient if the provided training annotation set reflects the wide range of structures present in the sets to be analyzed, our training sets compiled different sample types including mutant plants and abiotic treatments [38,43] in order to maximize variability of nucleus and chromocenter morphologies.

After settling a training set displaying a wide range of nuclear phenotypes, object annotation constituted the second critical step since DL algorithms are far from being deprived of human-like biases [45,46,47]. Consequently, the first step for preventing algorithm bias consists in reducing user-specific biases in the training set segmentation. In order to study inter-user differences using *Nucl.Eye.D*, the set of training images was annotated either manually by a single user (One_User) or by ten users (Ten_Users, each user analyzing a tenth of the images), or else by using *iCRAQ* by the same ten users (Ten_Users_*iCRAQ*). *Nucl.Eye.D* was released as a pipeline composed of three successive U-net neuronal networks [48] (Figure 5).

The release of a binary segmentation mask by *Nucl.Eye.D* relies on an uncertainty heatmap, with intensities ranging from 0 to 1 according to the certainty of the pixel to be part of the target object (Figure S2). Thus, in order to obtain a binary mask, a threshold needs to be set and is chosen by trial-and-error process, until the segmentation fits the best with the users’ expectations. However, to minimize human decision bias, the threshold is set to 0.5 by default. In this case, as soon as the model reaches a higher probability for a pixel to be defined as part of the object rather than the background, the pixel is kept within the segmentation. Training of the three successive models takes around 12 h (about 192,000 images, after data augmentation).

Once trained, models can be used to predict nuclear and chromocenter structures on any unannotated images. Upon the prediction process, input images are also refined into image fragments, with one nucleus per image, and a full-image prediction mask is automatically reconstructed from the different image fragments. This user-friendly output format allows masks to be overlaid to the original input images for calculating desired parameters (areas, signal intensities and shapes; Figure 5). In contrast to the training process, prediction of large datasets using the trained model can be performed within a few minutes (as an example, one image per second).

### 2.3. Nucl.Eye.D-Based Analysis of Nucleus and Chromocenters

**Figure 6:**
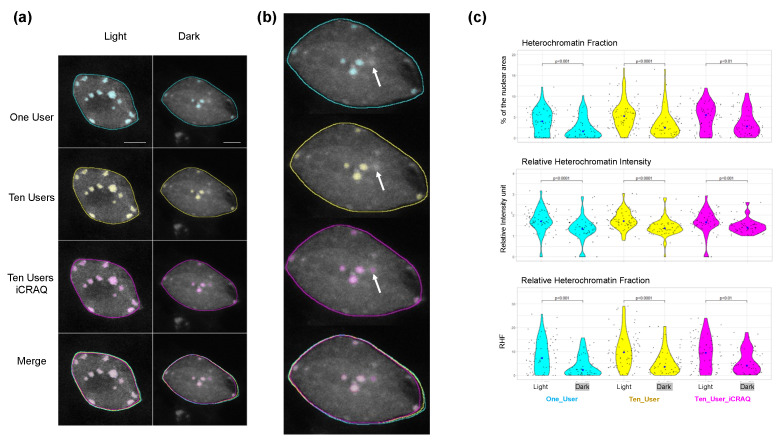
Comparison of nucleus and chromocenter segmentation between *Nucl.Eye.D* trained with manual or *iCRAQ* segmented image sets. (**a**) The same nuclei from the Light/Dark set used in Figure 2 were segmented by the three different *Nucl.Eye.D* segmentation approaches. The One_User *Nucl.Eye.D* was trained with an image set manually segmented by a single user. The Ten_User pipeline was trained with the same image set divided in ten image batches, each of them being manually segmented by a different user. The Ten_User_*iCRAQ* model was trained similarly to Ten_User but using the *iCRAQ* tool instead of manual segmentation. The borders of nucleus segmentation masks are shown as lines and chromocenter masks are overlayed on the images. To help comparisons, segmentation of the same nuclei performed by User_3 who produced the training set for the One_User *Nucl.Eye.D* model is shown. In addition, the merge of manual and *iCRAQ* segmentation by three users is shown. Merged images show the overlap between masks, comparing the One_User, Ten_Users and Ten_User_*iCRAQ Nucl.Eye.D* models. Scale bar = 5 μm. (**b**) Higher magnification of the overlap between masks obtained by One_User, Ten_Users and Ten_User_*iCRAQ Nucl.Eye.D* models. White arrow shows a CC segmented only by the Ten_User_*iCRAQ Nucl.Eye.D* model. (**c**) Distribution of HF, RHI and RHF of the nuclei from the Light/Dark Set (at least 50 nuclei per condition). Each black dot represents the measure for one nucleus. The large dot shows the median value. The *p* values were calculated according to the Mann–Whitney–Wilcoxon test.

We first used a Light/Dark dataset (Figure 5) to evaluate the accuracy of nucleus and chromocenter segmentation using *Nucl.Eye.D*. This Light/Dark set was not part of the training set and was newly produced independent using biological replicates, sample preparation, and image acquisition protocols to reflect variable laboratory conditions wherein one can use a given version of trained *Nucl.Eye.D* for analyzing its own data. As shown in Figure 6A, segmentation performed by *Nucl.Eye.D* shows a coherent overlay among different training sets. Whereas the One_User and Ten_Users models lead to relatively similar results, the Ten_Users_*iCRAQ* model recognized a few more chromocenters in Dark nuclei (Figure 6B and Figure S3).

**Figure 7:**
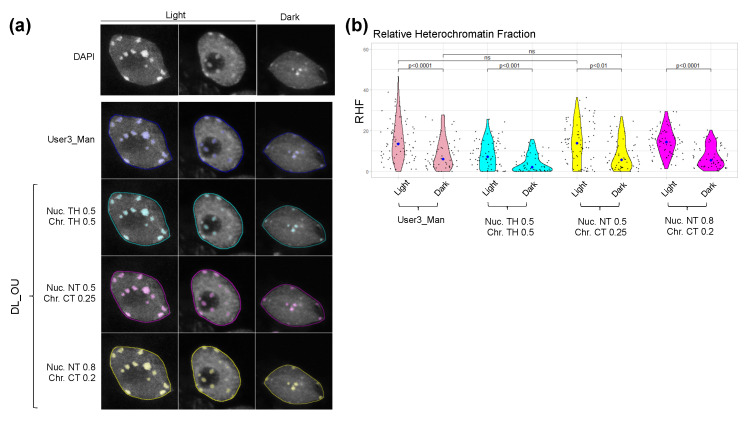
Segmentation of nuclei and chromocenters using different threshold settings in *Nucl.Eye.D*. (**a**) Nuclei from the Light/Dark set were segmented by the One_User *Nucl.Eye.D* pipeline that was trained with an image set manually segmented by User3. Manual segmentation of User3 is shown as comparison. The prediction was performed using three different couple values of threshold settings for nucleus/chromocenter: 0.5/0.5, 0.5/0.25 and 0.8/0.2. Borders of the nucleus segmentation masks are shown as lines and chromocenter masks as transparent color overlays. Scale bar = 5 μm. (**b**) RHF of nuclei from the Light/Dark Set (at least 50 nuclei analyzed) for the three different couple values of threshold settings used with the One_User *Nucl.Eye.D* pipeline and for the manual segmentation of User 3. Each black dot represents the measure for one nucleus. The large dot shows the median value. *p* values were calculated according to the Mann–Whitney–Wilcoxon test.

When calculating HF, RHI and RHF using the segmentation masks produced by *Nucl.Eye.D*, lower values of these features were expectedly observed in Dark nuclei, independently of the training set initially used (Figure 6A). Mean RHF values range from 8% to 10% and 4% to 6% in Light and Dark nuclei, respectively (Figure 6A). This indicates that chromocenter area or number predominantly influence RHF (Figure 3 and Figure S1). This may also reflect a high uncertainty for chromocenter prediction of the Light/Dark set, which may be linked to differences in sample preparation or image acquisition between the training and analyzed image sets. However, a close overlap with the results obtained with the manual segmentation was reached when defining a lower threshold for the chromocenter model (Figure 7).

**Figure 8:**
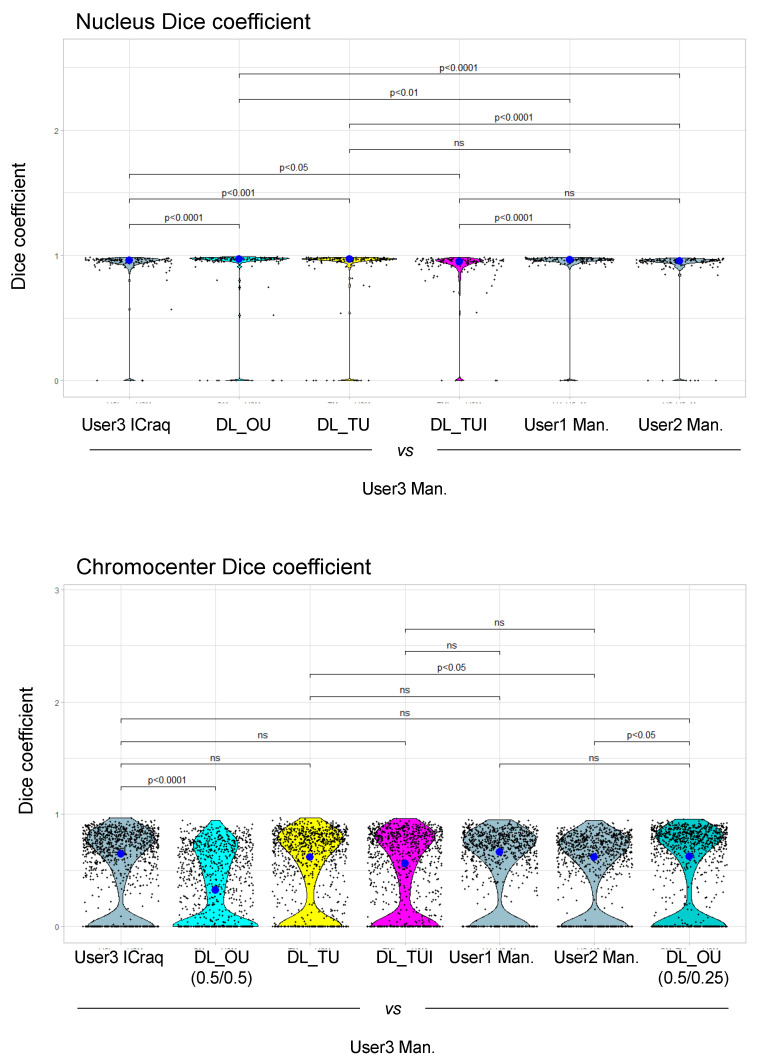
Dice coefficient calculated in between *Nucl.Eye.D*-based segmentation masks from different training sets. Segmentation masks obtained from different segmentation methods are compared with manually drawn segmentation masks from User 3. User 3 performed the segmentation of the training set for the One_User model, which serves here as reference. Statistical comparison was performed in between the segmentation methods according to the Mann–Whitney–Wilcoxon test. Each dot represents the measure for one object. The big dot shows the median value. n > 50 for nuclei and n > 400 for chromocenters.

To further document variability in object detection depending on the segmentation method, *iCRAQ*, manual or DL approaches were compared to the manual segmentation generated by User3 who segmented the One_User training set. Accuracy of the segmentation method was evaluated using the Dice coefficient (Figure 8). This analysis shows that, for nuclei segmentation, DL-based methods exhibit a high Dice coefficient with the segmentation masks of User 3 (Figure 8). Additionally, DL approaches display reduced variability as compared to most inter user or inter-method comparisons (Figure 8). The One_Users model, in which the training set was built by User3, shows a significantly higher Dice coefficient in comparison to the inter-method (User3 Icraq) and inter-user (User1 Man., User2 Man.) comparisons (Figure 8). This result can notably be explained by the characteristic of the Ten_Users models in which the specific traits of the objects have been learned by the detection convergence of ten people, thus reducing the personal biases of each user.

For chromocenters, the Dice coefficients only slightly vary between methods and users (Figure 8). DL-based chromocenter segmentation shows a comparable or slightly higher Dice coefficients when compared to inter-user or inter-method comparisons. Importantly, applying the nucleus/chromocenter 0.5/0.25 threshold used in the One_User model largely improves the Dice coefficient as compared to the 0.5/0.5 threshold, indicating the importance of fine-tuning these parameters for accurate segmentation (Figure 8).

Although DL efficiently reduces inter-user differences, thus providing ground for more powerful analysis of subtle changes, assisted segmentation by *iCRAQ* or any software is biased in the set of measurable features. While DL methods also suffer from the same drawbacks, the space of distinctive features they based their decision on is much larger and allows them to theoretically outperform any assisting software.

### 2.4. Nucl.Eye.D Analysis of the Ddm1 Dataset

**Figure 9:**
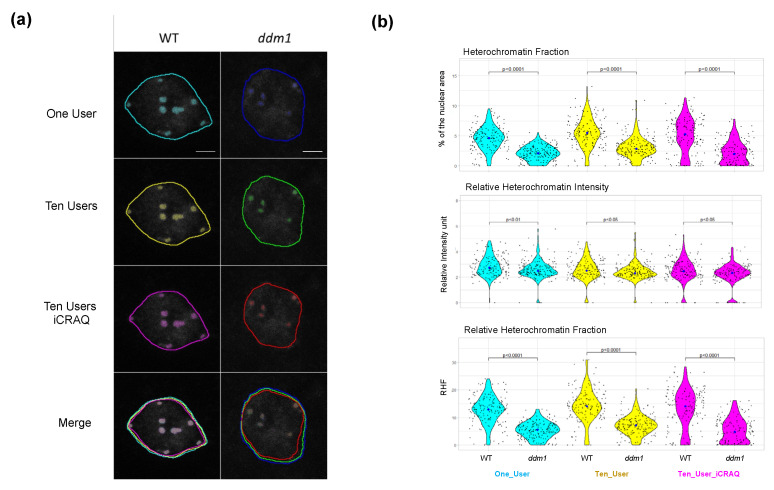
Segmentation of *ddm1* nuclei and chromocenters using *Nucl.Eye.D*. (**a**) DAPI-stained leaf nuclei from WT and *ddm1* plants were segmented by the three different *Nucl.Eye.D* pipelines: One_User, Ten_User and Ten_User_*iCRAQ*. Merged images show the overlap between masks, comparing the different pipelines. Scale bar = 5 μm. (**b**) Distribution of HF, RHI and RHF in a population of at least 150 nuclei per genotype. Each black dot represents the measure for one nucleus. The large dot shows the median value. *p* values were calculated according to a Mann–Whitney–Wilcoxon test.

In order to confirm the ability of *Nucl.Eye.D* to measure altered or non-canonical heterochromatin features, we used mutant Arabidopsis plants for *DDM1* These exhibit more pronounced alterations of heterochromatin patterns than the dark-grown plants [35,43,49]. We used the three different *Nucl.Eye.D* models to automatically segment a dataset of more than 150 wild-type (WT) and 150 *ddm1* nuclei, prepared following the same procedure as the one used to produce the training images. As shown in Figure 9A, the One_User, Ten_Users and Ten_Users_*iCRAQ* pipelines produced a coherent segmentation of low contrasted nuclei and small atypical *ddm1* chromocenters (Figure S4).

The three *Nucl.Eye.D* pipelines allow for detecting chromocenter morphology and accurately reporting the well-described defects of the *ddm1* mutant [43,49] (Figure 9B). The mean RHF in WT plants ranges between 12.5 and 14% [43,50], whereas *ddm1* nuclei exhibit an expected mean RHF from 5 to 8% [43] with reduced area of both nucleus and CC (Figure 7 and Figure 9). Analysis of this image set demonstrates the performance of *Nucl.Eye.D* for fast nuclei and chromocenter segmentation to identify significant differences in nucleus morphologies and phenotypes.

## 3. Materials and Methods

### 3.1. Plant Material and Growth Conditions

For the training set, wild-type (WT) Col-0 and ddm1-2 [43] plants were grown in vitro on solid GM medium (MS salts (Duchefa), 1% sucrose, 0.8% Agar-agar ultrapure (Merck), pH 5.8) in a culture chamber under a 16 h light (light intensity ∼150 μmol·m^−2^·s^−1^; 21 °C) and 8 h dark (19 °C) photoperiod.

For the Dark/Light set, seeds from wild-type (WT) Col-0 arabidopsis plants were surface-sterilized, plated on filter papers lying on MS medium supplemented with 0.9% agar and exposed to either a 16-/8-h (23/19 °C) white light/dark photoperiod or constant dark conditions (wrapped in 3 layers of aluminum foil). White light is generated by fluorescent bulbs (100 μmol·m^−2^·s^−1^). Seedlings are harvested under light condition or under safe green light for the dark condition [35].

### 3.2. Tissue Fixation and Nuclei Preparation for the Training Set

Leaves 3 and 4 from 21-day-old wild-type (WT) Col-0 and *ddm1-2* plants were washed 4 times (4 °C), at least 5 min, in fixative solution (3:1 ethanol/acetic acid; vol/vol). Leaves nuclei were extracted by chopping fixed tissue in LB-01 Buffer (15 mM Tris-HCl pH 7.5, 2 mM EDTA, 0.5 mM spermine, 80 mM KCl, 29 mM NaCl, 0.1% Triton X-100) with a razor blade. The nuclei containing solution was filtered through 20 µm nylon mesh and centrifugated 1 min (1000 g). Supernatant was spread on poly-lysine slides (Thermo Scientific, Waltham, MA, USA) and post fixation was performed using a 1:1 acetone/methanol (vol/vol) solution for 2 min. Slides were washed with Phosphate Buffer Saline x1 and incubated for 1 h at room temperature in permeabilization buffer (8% BSA, 0.01% Triton-X in Phosphate Buffer Saline × 1). Finally, 15 μL of Fluoromount-G (Southern Biotechnology CAT NO 0100–01) with 2 μg/mL 4′,6-Diamidino-2-phenylindol (DAPI) were added as mounting solution before deposing the coverslip. Image acquisition was performed on a Zeiss LSM 780 confocal microscope using an objective Plan-Aprochromat 63×/1.4 Oil DIC M27. Then, 405 nm laser excitation wavelength is used for DAPI. Emission is measured between 410 nm and 585 nm wavelength each image acquisition consisted in a Z-stack capture. Col0/*ddm1* images were acquired using the following settings: pictures were 0.1 × 0.1 × 0.43 µm/averaging by mean: 4/scan speed: 8. For training set, different gain and slice distances were used to diversify the set. All images are available at [41].

### 3.3. Tissue Fixation and Nuclei Preparation of Dark/Light Test Set

Seedlings were fixed in 4% formaldehyde for 3 h under the light or dark condition, and treated with a solution containing 0.5% cellulose Onozuka R10 (Yakult, Tokyo, Japan), 0.25% macerozyme R10 (Yakult), and 0.1% Triton X-100 for 1 h 30 min. Cotyledons were isolated and squashed on a glass slide, flash frozen in liquid nitrogen, and incubated with PEMSB (50 mM Pipes pH 7.3, 5 mM EGTA pH 7.1, 5 mM MgSO4, 0.05% saponin, 5% (wt/vol) BSA) before being mounted with Vectashield (Vector laboratories) supplemented with 2 μg·mL−1 DAPI (4′,6′-diamidino-2-phenylindole). Images were acquired using a confocal laser-scanning microscope (SP5, Leica). All confocal pictures used were 0.05 × 0.05 × 0.35 µm. The objective 63× with a numerical aperture of 1.40 was used, a zoom factor of 4.8 and acquired in 16 bit, 1024 × 1024. All images are available at [41].

### 3.4. Mask Preparation

Manual segmentation of nuclei and chromocenters was performed on ImageJ using the freehand tool and converted into binary masks. Image names were randomized prior to annotation. For training set #1, the segmentation was performed by a single user. For the training sets #2 and #3, 10 users each segmented 10% of the total set of images either manually (set #2) or using the *iCRAQ* tool (set #3).

### 3.5. iCRAQ Analysis

*iCRAQ* is a tool written in ImageJ macro language that relies on the *FeatureJ* (http://imagescience.org/meijering/software/featurej/, accessed on 1 January 2022) and Interactive H_Watershed (https://imagej.net/plugins/interactive-watershed, accessed on 1 January 2022) plugins; here, we used a version adapted from [40] to annotate images. Nuclei were detected via global thresholding of the median filtered z-projection (either standard deviation or maximum intensity) of the stack and the corresponding regions were saved as ImageJ regions of interest (ROIs). Incorrectly detected nucleus ROIs were suppressed manually. Likewise, missed nuclei were added manually. The input stack was cropped around each nucleus ROI. For chromocenter segmentation, the largest 3D structure tensor eigenvalue was calculated using the *FeatureJ* plugin, and its z-projection served as an input for the interactive H-watershed plugin. Image regions labeled as chromocenters were also saved as ROIs. Chromocenter ROIs could also be manually added or removed. Finally, binary masks of nucleus and chromocenter ROIs were used to produce an annotated image with three gray levels: 0 for the background, 128 for the nucleus and 255 for the chromocenters.

### 3.6. Nucl.Eye.D

The *Nucl.Eye.D* script was written in python using Keras and TensorFlow libraries for Neuronal network designing. U-net networks were built according to the original paper from Olaf Ronneberger [48]. Model training was performed using Google Collab allocated a Cuda v 11.2; Tesla P100-16 Go HBM2 GPU and Intel(R) Xeon(R) CPU @ 2.20 GHz CPU [40]. Full script, images and trained models are available in [41]. Importantly, training set was performed using images captured from tissue fixed with either formaldehyde or ethanol:acetic acid.

### 3.7. Morphometric Parameters Measurements

Each image acquisition consisted in a Z-stack capture with either a 0.35 or 0.43 μm slice distance, and the image was reconstructed using the z max plugin of ImageJ.

- Relative CC area, also called relative CC area fraction (RAF): area of each CC/nucleus area
- Heterochromatin fraction (HF): sum of all chromocenters’ areas/nucleus area
- Relative heterochromatin intensity (RHI): mean intensity of CC/mean intensity of nucleus
- Relative heterochromatin fraction (RHF): HF × RHI

### 3.8. Data Display and Statistics

Violin plots and statistics (Mann–Whitney–Wilcoxon test) were performed with RStudio, using ggplot2 [51].

## 4. Conclusions

Our work describes user-specific issues in manual nucleus and chromocenter detection and proposes improved segmentation tools: the semi-automatic ImageJ plug-in *iCRAQ* and the DL-based tool *Nucl.Eye.D*. The central motivation of this work was to test and provide plug-in assisted methods, which can be used to facilitate the production of training datasets to build a fully automated DL tool.

Both tools reduce the time of analysis and inter-user variability. *iCRAQ* can be easily implemented on a local computer (downloadable from [40] with a demonstration guide), and *Nucl.Eye.D* can be used directly online on a dedicated Google Collab environment to produce the binary masks [41]. The masks are then treated on ImageJ with a dedicated combination of macro [41] to compute the different nuclear and chromocenter morphometric parameters. In recent years, unsupervised learning techniques such as contrastive learning improved, especially for segmentation of medical images [52,53]. These have the benefit of needing much less (semi-supervised training) or no annotated images (self-supervised/unsupervised training) [53]. Consequently, these methods also reduce the risk of inducing a bias through the segmentation method used to build training set. However, to which extent these more recently developed methods can outperform the established CNN models for the segmentation of nuclei and subnuclear structures remains to be evaluated.

Although *Nucl.Eye.D* was trained here with a dataset composed of about 300 images, it provides accurate segmentation of nuclei and chromocenters, even on images produced from different protocols witnessing its adaptability. *Nucl.Eye.D* as DL-based approach for segmentation of nuclear and subnuclear structures should provide an interesting leap in the field of plant cytogenetics, and complete the existing range of DL-based tools already existing for phenotypic analyses in organs [28], leaves [27] or individual cells [29].

In contrast to tools such as *DeepImage J* [25] and *cellPose* [54], *Nucl.Eye.D* easily enables the training with user-specific datasets and purposes, and provides a ready to use workflow for object segmentation tasks inside ROI, without the need of self-building a workflow as previously proposed by ZerocostDL4Mic [55], providing a larger choice of combinable models.

Our methodology assists nuclei and subnuclear structure segmentation, possibly encouraging biologists to include DL-based methods to minimize human-derived biases in quantitative approaches of nucleus imaging.

Collectively, our study highlights that algorithm- and DL-based tools are not free of human biases introduced during the training process when it results from image choice and object segmentation. The “programmer”-based bias starts to be investigated as a potential explanation for dataset-specific performance [45,56]. Additionally, according to our thoughts, the growing trend of automatizing image segmentation and analysis should be accompanied by substantial efforts in assessing inter-user variability of the segmentation method [57]. We strongly recommend using DL based tools to enhance reproducibility (in the case that the dataset is large enough, we further recommend to retrain the DL tool on your own data, which then will be facilitated by *iCRAQ*).

In perspective, *Nucl.Eye.D* should contribute to expend the use of a DL-based approach in chromatin biology, offering the possibility to segment any subnuclear structures revealed by FISH- or immuno-staining (e.g., histone post-translational modifications, histone variants or chromatin binding factors).

## Supplementary Materials

The following supporting information can be downloaded at: https://www.mdpi.com/article/10.3390/epigenomes6040034/s1, Figure S1: Comparison of nuclear and chromocenter parameters quantified by different users and segmentation methods. Figure S2: From prediction to the comparison of binary segmentation masks Figure S3: Comparison of nuclear and chromocenter parameters quantification using NucleEyeD on Dark/Light image dataset.; Figure S4: Comparison of nuclear and chromocenter parameters quantification using NucleEyeD on a WT/ddm1 image dataset.

## References

[1] Weigel A.V., Chang C.-L., Shtengel G., Xu C.S., Hoffman D.P., Freeman M., Iyer N., Aaron J., Khuon S., Bogovic J. (2021). ER-to-Golgi Protein Delivery through an Interwoven, Tubular Network Extending from ER. Cell.

[2] Keuenhof K.S., Larsson Berglund L., Malmgren Hill S., Schneider K.L., Widlund P.O., Nyström T., Höög J.L. (2022). Large Organellar Changes Occur during Mild Heat Shock in Yeast. J. Cell Sci..

[3] Steblyanko Y., Rajendraprasad G., Osswald M., Eibes S., Jacome A., Geley S., Pereira A.J., Maiato H., Barisic M. (2020). Microtubule Poleward Flux in Human Cells Is Driven by the Coordinated Action of Four Kinesins. EMBO J..

[4] Colombo F., Norton E.G., Cocucci E. (2021). Microscopy Approaches to Study Extracellular Vesicles. Biochim. Biophys. Acta Gen. Subj..

[5] Van Treeck B., Parker R. (2019). Principles of Stress Granules Revealed by Imaging Approaches. Cold Spring Harb. Perspect Biol..

[6] Desset S., Poulet A., Tatout C., Bemer M., Baroux C. (2018). Quantitative 3D Analysis of Nuclear Morphology and Heterochromatin Organization from Whole-Mount Plant Tissue Using NucleusJ. Plant Chromatin Dynamics: Methods and Protocols.

[7] Mikulski P., Schubert D. (2020). Measurement of Arabidopsis Thaliana Nuclear Size and Shape. Methods Mol. Biol..

[8] Pavlova P., van Zanten M., Snoek B.L., de Jong H., Fransz P. (2021). 2D Morphometric Analysis of Arabidopsis Thaliana Nuclei Reveals Characteristic Profiles of Different Cell Types and Accessions. Chromosome Res..

[9] Arpòn J., Sakai K., Gaudin V., Andrey P. (2021). Spatial Modeling of Biological Patterns Shows Multiscale Organization of Arabidopsis Thaliana Heterochromatin. Sci. Rep..

[10] Simon L., Voisin M., Tatout C., Probst A.V. (2015). Structure and Function of Centromeric and Pericentromeric Heterochromatin in Arabidopsis Thaliana. Front. Plant Sci..

[11] Fransz P., De Jong J.H., Lysak M., Castiglione M.R., Schubert I. (2002). Interphase Chromosomes in Arabidopsis Are Organized as Well Defined Chromocenters from Which Euchromatin Loops Emanate. Proc. Natl. Acad Sci. USA.

[12] Almouzni G., Probst A.V. (2011). Heterochromatin Maintenance and Establishment: Lessons from the Mouse Pericentromere. Nucleus.

[13] Schroeder A.B., Dobson E.T.A., Rueden C.T., Tomancak P., Jug F., Eliceiri K.W. (2021). The ImageJ Ecosystem: Open-Source Software for Image Visualization, Processing, and Analysis. Protein Sci..

[14] Landini G., Randell D.A., Fouad S., Galton A. (2017). Automatic Thresholding from the Gradients of Region Boundaries. J. Microsc..

[15] Zhang J., Li C., Rahaman M.M., Yao Y., Ma P., Zhang J., Zhao X., Jiang T., Grzegorzek M. (2021). A Comprehensive Review of Image Analysis Methods for Microorganism Counting: From Classical Image Processing to Deep Learning Approaches. Artif. Intell. Rev..

[16] Hunt G.J., Dane M.A., Korkola J.E., Heiser L.M., Gagnon-Bartsch J.A. (2020). Automatic Transformation and Integration to Improve Visualization and Discovery of Latent Effects in Imaging Data. J. Comput. Graph. Stat..

[17] Dubos T., Poulet A., Gonthier-Gueret C., Mougeot G., Vanrobays E., Li Y., Tutois S., Pery E., Chausse F., Probst A.V. (2020). Automated 3D Bio-Imaging Analysis of Nuclear Organization by NucleusJ 2.0. Nucleus.

[18] Dubos T., Poulet A., Thomson G., Péry E., Chausse F., Tatout C., Desset S., van Wolfswinkel J.C., Jacob Y. (2022). NODeJ: An ImageJ Plugin for 3D Segmentation of Nuclear Objects. BMC Bioinform..

[19] Sensakovic W.F., Starkey A., Roberts R., Straus C., Caligiuri P., Kocherginsky M., Armato S.G. (2010). The Influence of Initial Outlines on Manual Segmentation. Med. Phys..

[20] Renard F., Guedria S., Palma N.D., Vuillerme N. (2020). Variability and Reproducibility in Deep Learning for Medical Image Segmentation. Sci. Rep..

[21] Seeland M., Rzanny M., Boho D., Wäldchen J., Mäder P. (2019). Image-Based Classification of Plant Genus and Family for Trained and Untrained Plant Species. BMC Bioinform..

[22] Krull A., Buchholz T.-O., Jug F. Noise2Void—Learning Denoising From Single Noisy Images. Proceedings of the 2019 IEEE/CVF Conference on Computer Vision and Pattern Recognition (CVPR).

[23] Zhou W., Yang Y., Yu C., Liu J., Duan X., Weng Z., Chen D., Liang Q., Fang Q., Zhou J. (2021). Ensembled Deep Learning Model Outperforms Human Experts in Diagnosing Biliary Atresia from Sonographic Gallbladder Images. Nat. Commun..

[24] Godec P., Pančur M., Ilenič N., Čopar A., Stražar M., Erjavec A., Pretnar A., Demšar J., Starič A., Toplak M. (2019). Democratized Image Analytics by Visual Programming through Integration of Deep Models and Small-Scale Machine Learning. Nat. Commun..

[25] Gómez-de-Mariscal E., García-López-de-Haro C., Ouyang W., Donati L., Lundberg E., Unser M., Muñoz-Barrutia A., Sage D. (2021). DeepImageJ: A User-Friendly Environment to Run Deep Learning Models in ImageJ. Nat. Methods.

[26] Shepley A., Falzon G., Lawson C., Meek P., Kwan P. (2021). U-Infuse: Democratization of Customizable Deep Learning for Object Detection. Sensors.

[27] Atanbori J., French A.P., Pridmore T.P. (2020). Towards Infield, Live Plant Phenotyping Using a Reduced-Parameter CNN. Mach. Vis. Appl..

[28] Yasrab R., Atkinson J.A., Wells D.M., French A.P., Pridmore T.P., Pound M.P. (2019). RootNav 2.0: Deep Learning for Automatic Navigation of Complex Plant Root Architectures. Gigascience.

[29] Li S., Li L., Fan W., Ma S., Zhang C., Kim J.C., Wang K., Russinova E., Zhu Y., Zhou Y. (2022). LeafNet: A Tool for Segmenting and Quantifying Stomata and Pavement Cells. Plant Cell.

[30] Li J., Peng J., Jiang X., Rea A.C., Peng J., Hu J. (2021). DeepLearnMOR: A Deep-Learning Framework for Fluorescence Image-Based Classification of Organelle Morphology. Plant Physiol..

[31] Tatout C., Mougeot G., Parry G., Baroux C., Pradillo M., Evans D. (2022). The INDEPTH (Impact of Nuclear Domains On Gene Expression and Plant Traits) Academy—A Community Resource for Plant Science. J. Exp. Bot..

[32] Brändle F., Frühbauer B., Jagannathan M. (2022). Principles and Functions of Pericentromeric Satellite DNA Clustering into Chromocenters. Semin. Cell Dev. Biol..

[33] Goto C., Hara-Nishimura I., Tamura K. (2021). Regulation and Physiological Significance of the Nuclear Shape in Plants. Front. Plant Sci..

[34] Pecinka A., Chevalier C., Colas I., Kalantidis K., Varotto S., Krugman T., Michailidis C., Vallés M.-P., Muñoz A., Pradillo M. (2020). Chromatin Dynamics during Interphase and Cell Division: Similarities and Differences between Model and Crop Plants. J. Exp. Bot..

[35] Bourbousse C., Mestiri I., Zabulon G., Bourge M., Formiggini F., Koini M.A., Brown S.C., Fransz P., Bowler C., Barneche F. (2015). Light Signaling Controls Nuclear Architecture Reorganization during Seedling Establishment. Proc. Natl. Acad Sci. USA.

[36] Benoit M., Layat E., Tourmente S., Probst A.V. (2013). Heterochromatin Dynamics during Developmental Transitions in Arabidopsis—a Focus on Ribosomal DNA Loci. Gene.

[37] Pecinka A., Dinh H.Q., Baubec T., Rosa M., Lettner N., Scheid O.M. (2010). Epigenetic Regulation of Repetitive Elements Is Attenuated by Prolonged Heat Stress in Arabidopsis. Plant Cell.

[38] Graindorge S., Cognat V., Johann to Berens P., Mutterer J., Molinier J. (2019). Photodamage Repair Pathways Contribute to the Accurate Maintenance of the DNA Methylome Landscape upon UV Exposure. PLoS Genet..

[39] Schindelin J., Arganda-Carreras I., Frise E., Kaynig V., Longair M., Pietzsch T., Preibisch S., Rueden C., Saalfeld S., Schmid B. (2012). Fiji: An Open-Source Platform for Biological-Image Analysis. Nat. Methods.

[40] Schivre G. (2022). ICRAQ. https://github.com/gschivre/iCRAQ.

[41] Johann to Berens P., Theune M. (2022). Nucl.Eye.D—Zenodo. https://zenodo.org/record/7075507.

[42] Vongs A., Kakutani T., Martienssen R.A., Richards E.J. (1993). Arabidopsis Thaliana DNA Methylation Mutants. Science.

[43] Soppe W.J.J., Jasencakova Z., Houben A., Kakutani T., Meister A., Huang M.S., Jacobsen S.E., Schubert I., Fransz P.F. (2002). DNA Methylation Controls Histone H3 Lysine 9 Methylation and Heterochromatin Assembly in Arabidopsis. EMBO J..

[44] Li C.H., Tam P.K.S. (1998). An Iterative Algorithm for Minimum Cross Entropy Thresholding. Pattern Recognit. Lett..

[45] Cazes M., Franiatte N., Delmas A., André J.-M., Rodier M., Kaadoud I.C. Evaluation of the Sensitivity of Cognitive Biases in the Design of Artificial Intelligence. Proceedings of the Rencontres des Jeunes Chercheurs en Intelligence Artificielle (RJCIA’21) Plate-Forme Intelligence Artificielle (PFIA’21).

[46] Alzubaidi L., Zhang J., Humaidi A.J., Al-Dujaili A., Duan Y., Al-Shamma O., Santamaría J., Fadhel M.A., Al-Amidie M., Farhan L. (2021). Review of Deep Learning: Concepts, CNN Architectures, Challenges, Applications, Future Directions. J. Big Data.

[47] Paullada A., Raji I.D., Bender E.M., Denton E., Hanna A. (2021). Data and Its (Dis)Contents: A Survey of Dataset Development and Use in Machine Learning Research. Patterns.

[48] Ronneberger O., Fischer P., Brox T. (2015). U-Net: Convolutional Networks for Biomedical Image Segmentation. MICCAI 2015. Lecture Notes in Computer Science.

[49] Mathieu O., Jasencakova Z., Vaillant I., Gendrel A.-V., Colot V., Schubert I., Tourmente S. (2003). Changes in 5S RDNA Chromatin Organization and Transcription during Heterochromatin Establishment in Arabidopsis. Plant Cell.

[50] Snoek B.L., Pavlova P., Tessadori F., Peeters A.J.M., Bourbousse C., Barneche F., de Jong H., Fransz P.F., van Zanten M. (2017). Genetic Dissection of Morphometric Traits Reveals That Phytochrome B Affects Nucleus Size and Heterochromatin Organization in Arabidopsis Thaliana. G3 Bethesda.

[51] RStudio Open Source & Professional Software for Data Science Teams—RStudio. https://www.rstudio.com/.

[52] Van Voorst H., Konduri P.R., van Poppel L.M., van der Steen W., van der Sluijs P.M., Slot E.M.H., Emmer B.J., van Zwam W.H., Roos Y.B.W.E.M., Majoie C.B.L.M. (2022). Unsupervised Deep Learning for Stroke Lesion Segmentation on Follow-up CT Based on Generative Adversarial Networks. Am. J. Neuroradiol..

[53] Wang K., Zhan B., Zu C., Wu X., Zhou J., Zhou L., Wang Y. (2022). Semi-Supervised Medical Image Segmentation via a Tripled-Uncertainty Guided Mean Teacher Model with Contrastive Learning. Med. Image Anal..

[54] Stringer C., Wang T., Michaelos M., Pachitariu M. (2021). Cellpose: A Generalist Algorithm for Cellular Segmentation. Nat. Methods.

[55] von Chamier L., Laine R.F., Jukkala J., Spahn C., Krentzel D., Nehme E., Lerche M., Hernández-Pérez S., Mattila P.K., Karinou E. (2021). Democratising Deep Learning for Microscopy with ZeroCostDL4Mic. Nat. Commun..

[56] Sun W., Nasraoui O., Shafto P. (2020). Evolution and Impact of Bias in Human and Machine Learning Algorithm Interaction. PLoS ONE.

[57] Robinson R., Valindria V.V., Bai W., Oktay O., Kainz B., Suzuki H., Sanghvi M.M., Aung N., Paiva J.M., Zemrak F. (2019). Automated Quality Control in Image Segmentation: Application to the UK Biobank Cardiovascular Magnetic Resonance Imaging Study. J. Cardiovasc. Magn. Reson..

