# Supplementary material for: Advanced Image Analysis Methods for Automated Segmentation of Subnuclear Chromatin Domains
Source: Epigenomes. 2022 Oct 5;6(4):34. doi: 10.3390/epigenomes6040034 (PMC9624336; doi:10.3390/epigenomes6040034)
Supplement: Supplementary file 1 [file epigenomes-06-00034-s001.zip › epigenomes-1814089-supplementary.pdf]

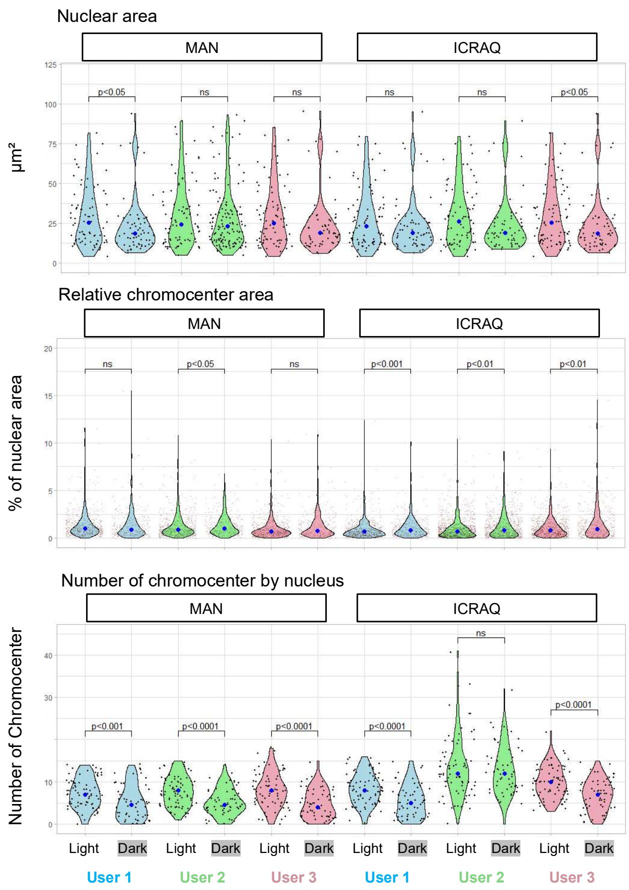

Supplemental Figure S1. Comparison of nuclear and chromocenter parameters quantified by different users and segmentation methods. Each black dot represents the measure for one nucleus. The large dot shows the median value. The p-values were calculated according to the Mann Whitney Wilcoxon test. n>50 nuclei per condition and user.

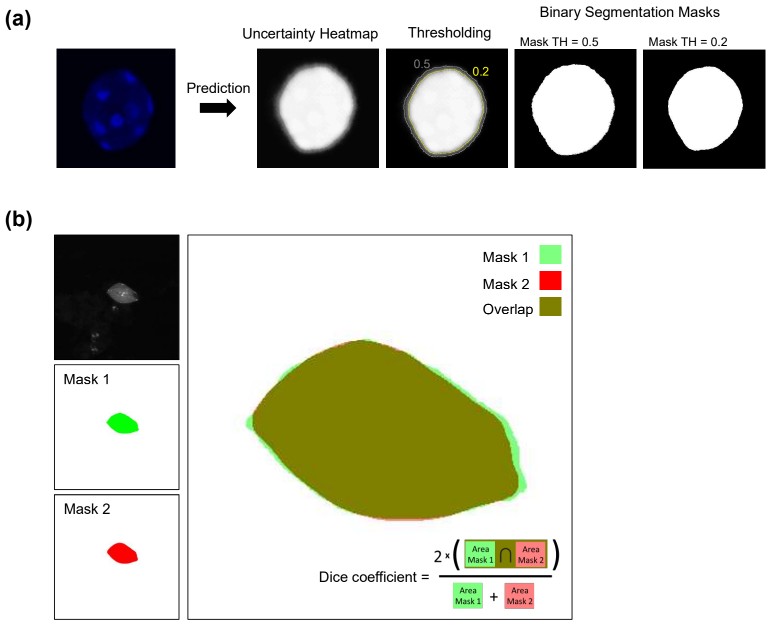

Supplemental Figure S2. From prediction to the comparison of binary segmentation masks. **(a)** Graphical representation of the predicted uncertainty heatmap and thresholding process to obtain binary segmentation masks. **(b)** Graphical representation of the overlap and caluclation of the Dice coefficient between binary segmentation masks.

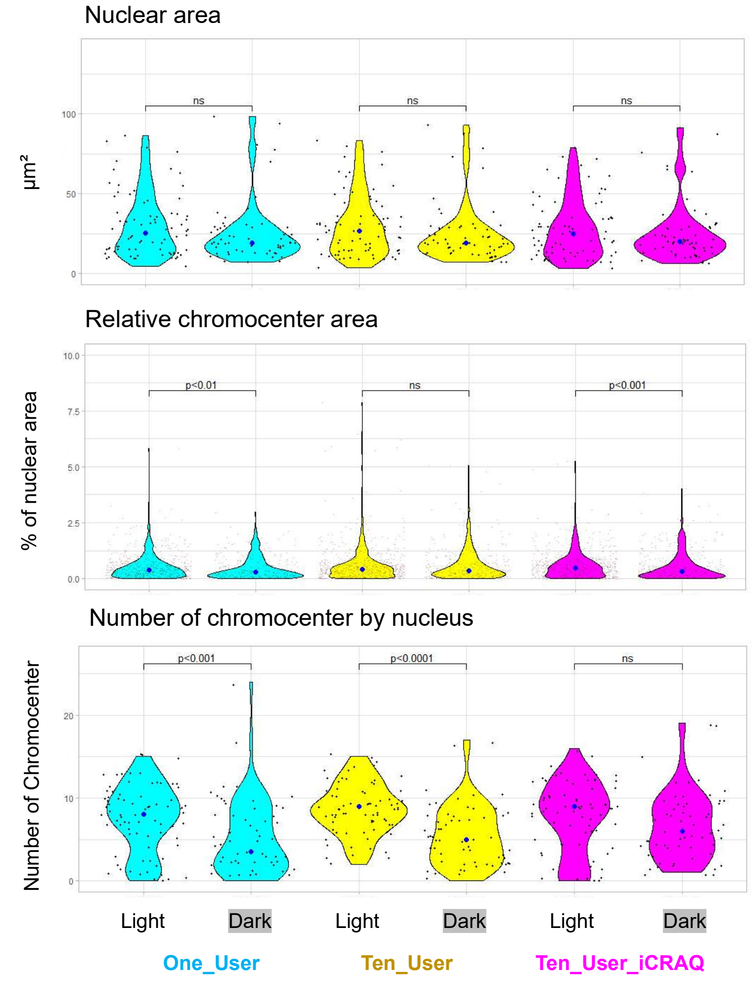

Supplemental Figure S3. Comparison of nuclear and chromocenter parameters quantification using *NucleEyeD* on Dark/Light image dataset. Violin plots illustrating the distribution of nuclear area, relative chromocenter area, and number of chromocenters. Each black dot represents the measure for one nucleus. The large dot shows the median value. The p-values were calculated according to the Mann Whitney Wilcoxon test. n>50 nuclei per condition.

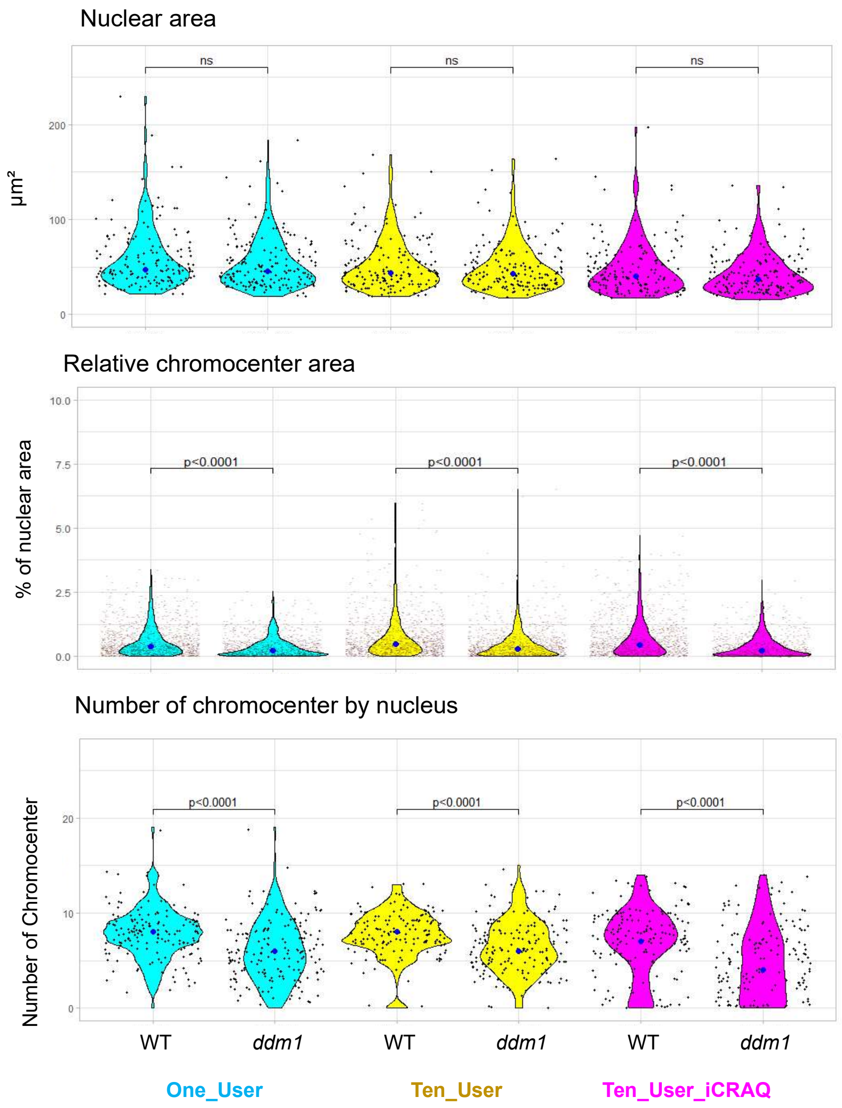

Supplemental Figure S4. Comparison of nuclear and chromocenter parameters quantification using *NucleEyeD* on WT/*ddm1* image dataset. Violin plots illustrating the distribution of nuclear area, relative chromocenter area and number of chromocenters. Each black dot represents the measure for one nucleus. The large dot shows the median value. The p-values were calculated according to the Mann Whitney Wilcoxon test.
